# Maternal dietary intake and physical activity habits during the postpartum period: associations with clinician advice in a sample of Australian first time mothers

**DOI:** 10.1186/s12884-016-0812-4

**Published:** 2016-02-01

**Authors:** Paige van der Pligt, Ellinor K Olander, Kylie Ball, David Crawford, Kylie D Hesketh, Megan Teychenne, Karen Campbell

**Affiliations:** Centre for Physical activity and Nutrition Research (C-PAN), Deakin University, Burwood Campus, 221 Burwood Highway, Burwood, VIC 3125 Australia; Centre for Maternal and Child Health Research, City University London, London, UK

**Keywords:** Pregnancy, postpartum, maternal, advice, health professional, diet, physical activity

## Abstract

**Background:**

Numerous health benefits are associated with achieving optimal diet and physical activity behaviours during and after pregnancy. Understanding predictors of these behaviours is an important public health consideration, yet little is known regarding associations between clinician advice and diet and physical activity behaviours in postpartum women. The aims of this study were to compare the frequency of dietary and physical activity advice provided by clinicians during and after pregnancy and assess if this advice is associated with postpartum diet and physical activity behaviours.

**Methods:**

First time mothers (n = 448) enrolled in the Melbourne InFANT Extend trial completed the Cancer Council of Australia’s Food Frequency Questionnaire when they were three to four months postpartum, which assessed usual fruit and vegetable intake (serves/day). Total physical activity time, time spent walking and time in both moderate and vigorous activity for the previous week (min/week) were assessed using the Active Australia Survey. Advice received during and following pregnancy were assessed by separate survey items, which asked whether a healthcare practitioner had discussed eating a healthy diet and being physically active. Linear and logistic regression assessed associations of advice with dietary intake and physical activity.

**Results:**

In total, 8.6 % of women met guidelines for combined fruit and vegetable intake. Overall, mean total physical activity time was 350.9 ± 281.1 min/week. Time spent walking (251.97 ± 196.78 min/week), was greater than time spent in moderate (36.68 ± 88.58 min/week) or vigorous activity (61.74 ± 109.96 min/week) and 63.2 % of women were meeting physical activity recommendations. The majority of women reported they received advice regarding healthy eating (87.1 %) and physical activity (82.8 %) during pregnancy. Fewer women reported receiving healthy eating (47.5 %) and physical activity (51.9 %) advice by three months postpartum. There was no significant association found between provision of dietary and/or physical activity advice, and mother’s dietary intakes or physical activity levels.

**Conclusions:**

Healthy diet and physical activity advice was received less after pregnancy than during pregnancy yet no association between receipt of advice and behaviour was observed. More intensive approaches than provision of advice may be required to promote healthy diet and physical activity behaviours in new mothers.

**Trial registration:**

Australian New Zealand Clinical Trials Registry (ACTRN12611000386932 13/04/2011)

## Background

Much evidence exists supporting the health benefits for women in achieving healthy lifestyle behaviours both during [1–5] and following [6–8] pregnancy. Adopting healthy dietary and physical activity behaviours during the postpartum period is particularly important to promote optimal maternal health, both in the short and long term. A diet adequate in fruit and vegetables and low in fat has been shown to play a vital role in reducing the risk for many diseases [8, 9] and research has linked poor dietary habits with the development of non–communicable diseases including type 2 diabetes, hypertension, cardiovascular disease and some cancers [10, 11]. Specifically following childbirth, healthy eating is important to adequately support breastfeeding [12] and demonstrate healthy role modelling behaviours for infants [12]. However it is commonplace for women of all ages not to meet dietary intake recommendations [13]. Although some women may eat healthily during pregnancy [14] these habits often discontinue following childbirth [14, 15] with declines in adequacy of fruit and vegetable intake having been observed [16–18].

Regular physical activity during the postpartum period has been associated with improved mental health [6, 7] as well as improved cholesterol levels and insulin sensitivity [6, 19], and increased chance of women returning to pre-pregnancy weight [6, 20, 21]. Further, physical activity has been shown to be a critical influence on maternal weight [22] and predicts postpartum weight retention (PPWR) 12 months after childbirth [22, 23]. In the interest of maternal health, a greater understanding of physical activity patterns during the postpartum period is required, yet the literature to date suggests an observed decline in physical activity following childbirth [5, 6, 24, 25].

Importantly, both PPWR and long term development and persistence of obesity in women [26–30] have been attributed to suboptimal maternal diet and physical activity behaviours. Results from systematic reviews of interventions aimed at limiting PPWR have consistently shown that the majority of successful studies have included both diet and physical activity intervention

components in their design [31]. Hence understanding factors which may promote healthy diet and physical activity behaviours is key to ensuring women receive the support they need to engage in these behaviours.

An important opportunity for provision of support lies with advice provided by antenatal healthcare clinicians. The antenatal period is a time in which women are in frequent contact with a wide range of health professionals [1, 14, 32], depending on their preferred choice of antenatal care. Obstetricians, general practitioners and midwives as well as other antenatal healthcare workers are routine providers of care within the antenatal system, which in some countries has been estimated to reach almost 100 % of the pregnant population [1, 32]. Moreover, health professionals are often viewed as authorities regarding maternal health information and advice both during [33, 34] and following pregnancy [35].

Compared to during pregnancy, less opportunity presents in the months following childbirth for women to be seen by healthcare practitioners. Formal, scheduled face-to-face practitioner and patient contact often occurs on fewer occasions during the postpartum period compared with during pregnancy, [36] and formal care is inconsistent between countries. In Australia for example, there are no standardised guidelines for the recommended frequency/schedule of routine postpartum visits to healthcare providers [37]. In the UK, however, formal guidelines such as the UK National Institute for Health and Care Excellence (NICE) recommend that diet and physical activity advice be provided to women following childbirth [38]. The NICE guidelines also recommend a greater number of antenatal appointments with a health professional during pregnancy compared with the postpartum period [38]. Yet, whilst some postpartum education is provided for women by nursing and other healthcare professionals, much of the support is focused on breastfeeding and care of the newborn, rather than the physical health of the mother [39]. It is thus not surprising that primary care services for women during the postpartum period have been described as being inconsistent, fragmented across disciplines and not adequate in meeting population needs [37, 40].

There is very little known about the diet and physical activity advice women receive during the postpartum period. A better understanding of the provision of advice and recommendations women receive and if these are associated with diet and physical activity behaviours is necessary to identify opportunities for provision of healthy lifestyle support for new mothers during this unique life stage. The aims of this study were to (i) compare the frequency of dietary and physical activity advice provided by clinicians during pregnancy and in the postpartum period and (ii) assess if advice received by women is associated with maternal postpartum diet and physical activity behaviours in a sample of first time mothers.

## Methods

### Participants

Participants were from the extended Melbourne Infant Feeding Activity and Nutrition Trial Program (InFANT Extend), a cluster-randomised controlled intervention trial which recruited first time mothers who attended first time parent groups at their local Maternal and Child Health Centres in Victoria. A two-stage random sampling design was used to select first time parent groups across all socio-economic areas. Areas eligible for local government area (LGA) selection were those areas which had not previously been involved in the earlier Melbourne InFANT trial (2008–2010) [41]. To ensure inclusion of a broad representation of socio- economic areas, each LGA was classified by socio-economic position (SEP) using the Australian Bureau of Statistics Socio-Economic Indices for Areas (SIEFA). Local Government areas with SEIFAs in the lowest quintile across the state were eligible. In the first stage, nine LGAs qualified to be approached for recruitment and seven agreed to take part.

In the second stage of recruitment, within the local government areas, 62 first-time parents groups were selected from the seven LGAs. For a group to be eligible, a minimum of eight participants were required to consent per first-time parent group within mid and higher SEP LGAs and six participants were required per first-time parent group within the lower SEP LGAs, as participation rates tend to be low in health behaviour interventions for vulnerable population groups or low socio-economic areas [42]. Parents needed to be English literate. When first-time parents groups declined to participate, another group was approached to take part. Written consent to participate was obtained and groups were then randomly allocated to intervention or control arms. The InFANT Extend trial was approved by the Deakin University \Human Research Ethics Committee (2011–029) (2007–175) (11/02/2011) and the Victorian Government Department of Human Services, Office for Children Research Co-ordinating Committee.

### Measures

This study reports baseline data completed when mothers were approximately 3 months postpartum. Questionnaires were distributed to all mothers at the recruitment visit when women consented to take part in the study. Demographic and socioeconomic variables included maternal age, marital status, birth country, education level, employment level, smoking status (currently and during pregnancy), breastfeeding status and weekly household income level.

#### Anthropometry

Pre-pregnancy weight was self-reported. Postpartum weight was measured by trained research staff when women were approximately 3 months postpartum. Weight in light clothing and without shoes was measured once using Tanita digital scales (Model 1582) and recorded to the nearest 0.01 kg. Height was measured using a Victar stadiometer. All equipment used for anthropometry measurements were calibrated prior to the beginning of the InFANT Extend intervention. Two measurements were taken separately and were recorded to the nearest 0.1 cm. If the two measurements disagreed by 0.5 cm or more, a third measurement was taken and recorded. The average of the height measurements was used to calculate maternal pre- pregnancy BMI and postpartum BMI, calculated as weight (kg) / height (m^2^). Body Mass Index classification was according to World Health Organization criteria (underweight (BMI <18.5), healthy weight (BMI 18.5 to <25.0), overweight (BMI 25.0 to <30.0) or obese (BMI ≥ 30.0)) [43].

#### Dietary intake

Dietary intake was assessed using the Cancer Council of Victoria’s Dietary Questionnaire for Epidemiological Studies (DQES) version 3.1 Food Frequency Questionnaire [44]. The DQES has been previously validated against seven day weighed food records [45, 46] and has been shown to be a useful assessment of dietary intake in the Australian population [46, 47]. The DQES assesses usual food consumption over the previous 12 months and comprises a food list of 74 items with 10 frequency response options assessing frequency of intake from ‘never’ to ‘3 or more times per day’ [44]. These data were converted into daily equivalent frequencies (DEFs) according the Cancer Council of Victoria’s protocol and divided into several different food categories, including non-core foods. Several separate items asked women to report their usual intake of specific types and quantities of different core and non-core foods. One item asked women to report their usual intake of fruit (serves per day); a separate item assessed intake of vegetables (excluding potatoes) (serves per day); and one item assessed intake of non-diet soft drink (glasses per day). A serving sizes guide was given to assist in quantification of intake of these items. Fruit and vegetable intake responses were compared with Australian adult recommendations for daily fruit and vegetable intake [48].

#### Physical activity

Postpartum physical activity (duration and intensity) was assessed using the Australian Institute of Health and Welfare’s Active Australia Survey (AAS) [49, 50]. The AAS has been used frequently to assess physical activity in Australian adult populations [51–53], with established test – retest reliability and validity in Australian [52, 54–56] and US [57] adult populations and deemed similar to that of the International Physical Activity Questionnaire [50, 51]. The AAS has been also been previously validated against accelerometers in Australian [56] and US women [57].

Women were asked to estimate the total duration (number of times and total hours and minutes) of time they spent walking continuously (for at least 10 minutes) for recreation, exercise or to get from place to place, in the week prior to completing the questionnaire. They were also asked to estimate the total duration (number of times and total hours and minutes) of time they spent in moderate and vigorous activities which excluded household chores, gardening or yard work. Total physical activity time (min/week) was calculated by summing the total time spent walking, time spent in moderate physical activity and twice the time spent in vigorous physical activity, as per AAS scoring protocols [50]. To avoid the possibility of errors due to over- reporting, data for each activity type were truncated at 840 min/week (14 h). Total time in all activities was truncated at 1680 min/week (28 h). Categorical variables were created to define ‘sufficient minutes’ of physical activity (≥150 minutes per week), ‘sufficient sessions’ (≥5 sessions per week) and ‘sufficient activity’ (≥150 minutes per week plus ≥ 5 sessions per week) [50]. Physical activity levels were defined as either meeting recommendations (i.e. sufficient activity) or not. Women were classified as being ‘sedentary’ if their combined total time spent walking, in moderate activity and in vigorous activity was equal to zero, as per the survey protocol.

#### Clinician advice

Provision of clinician advice during pregnancy was measured by a single item which asked women if during their pregnancy, a doctor, nurse or other healthcare practitioner talked with them about dietary advice: ‘eating a healthy diet during pregnancy’ and physical activity advice: ‘being physically active during pregnancy’. Provision of clinician advice during the postpartum period was measured by a single item which asked women if, since delivering their baby, they had they received advice from their doctor, nurse or other healthcare worker about dietary advice: ‘eating a healthy diet’; or physical activity: ‘being physically active’.

### Statistical analyses

Data were analysed using SPSS statistical package version 21, or for regression analyses assessing associations of clinician advice with maternal health behaviour, Stata Version 12 was used to allow controlling for clustering. Analyses included descriptive statistics to characterize the sample and distributions of key variables. Chi square tests were conducted to detect differences in the proportion of women who reported receiving advice during pregnancy compared to during the postpartum period. For associations of clinician advice with maternal diet and physical activity, linear regression was conducted when outcomes were continuous. Continuous outcome measures were checked for normality and when found to be non-normally distributed were transformed using root or log transformations. Binomial or multinomial logistic regression analyses were conducted when outcomes were categorical. Statistical significance was set as p <0.05 for all analyses.

Maternal age, education, income and BMI were adjusted for in the analysis when dietary and physical activity outcomes were assessed. Finally, clustering by first-time parents’ groups was accounted for in all models, using the ‘cluster by’ command in Stata. Adjustment for clustering within the analysis is necessary when assessing a cluster-randomised trial such as the Melbourne InFANT RCT [41] and accounts for the design effect and expected intra-cluster correlations of maternal characteristics within a first-time parent group.

## Results

Women were excluded from the analysis in this study if they were not first time mothers (n = 15), if they had non-singleton pregnancies (n = 6) or if they had not provided detail regarding parity (n = 6). Survey data were missing for 2 women, leaving data for 448 women included in the analysis. Characteristics of these women are presented in Table 1.

**Table 1:** Maternal characteristics

	Mean ± SD	Range
Maternal age (years)	31.9±4.25	19.3-43.5
Pre-pregnancy BMI (kg/m^2^) (n=430)	24.8±4.88	16.3-55.0
Postpartum BMI (kg/m^2^) (n=416)	26.2±5.00	17.1-46.8
	n (%)	
Marital status		
Married	74.1	
De facto	22.3	
Separated/Divorced	1.3	
Never married	2.2	
Birth country		
Australia	75.9	
UK	3.3	
New Zealand	2.2	
Other	18.5	
Weekly household income (n=433)		
$1-1499	36.7	
$1500-1999	22.0	
$2000 or more	29.7	
Unsure/Don’t want to answer	11.6	
Education (n=447)		
No qualification/up to year 12	11.6	
Trade/apprenticeship/certificate/diploma	30.0	
University degree/Higher degree	58.4	
Employment status (n=446)		
Full time work	3.4	
Part time work	6.3	
Keeping house/raising children full time	88.6	
Studying full time/Unemployed	1.8	
Smoking during pregnancy		
Yes	3.8	
No	96.2	
Smoking currently		
Yes	5.4	
No	94.7	
Breastfeeding		
Exclusive breastfeeding	49.6	
Breastfeeding combined with formula or other fluids	28.1	
Formula feeding only	22.3	

n=448 unless otherwise stated

### Dietary intake

Maternal dietary intakes are presented in Table 2. Overall, approximately half of all women (55.4 %) met fruit recommendations defined as 2 or more serves/day. 8.6 % of all women met recommendations for vegetable intake defined as 5 or more serves/day, with 44.9 % of women consuming 2 or fewer serves/day of vegetables [48]. Just 7.2 % of all women met recommendations for both fruit and vegetable intake. Approximately half (53.4 %) of all women reported consuming less than one glass of regular soft drink per day and approximately one third of all women (33.0 %) did not consume soft drink on a daily basis. Mean total non- core snack food DEF score, comprised of six non-core sweet and savoury snack foods, was 1.39 ± 1.18 and ranged from 0.02 – 10.85 DEF.

**Table 2:** Maternal postpartum dietary patterns

	n	(%)
Daily fruit intake (n=442)		
Zero or less than one serve	75	17.0
1 serve	123	27.8
2 serves	164	37.1
3 serves	66	14.9
4-6 serves	14	3.2
Fruit intake meeting recommendations^b^		
Yes	245	55.4
No	197	44.6
Daily vegetable intake (n=442)		
1 serve or less	79	17.8
2 serves	120	27.1
3 serves	147	33.3
4 serves	58	13.1
5 serves	26	5.9
6-7 serves	12	2.7
Vegetable intake meeting recommendations^b^		
Yes	38	8.6
No	404	91.4
Combined fruit and vegetable intake meeting recommendations		
Yes	32	7.2
No	410	92.8
Daily soft drink intake (n=442)		
None	146	33.0
Less than one glass	236	53.4
1 glass or more	60	13.7
	Mean ± SD	Range
Non core snack food intake score total (DEF)^a^ (n=442)	1.39±1.18	0.02-10.85
Chocolate or confectionary containing chocolate	0.42±0.47	0.00-3.00
Confectionary	0.21±0.33	0.00-3.00
Ice cream	0.18±0.23	0.00-2.00
Cakes or sweet pastries	0.19±0.21	0.00-2.00
Sweet biscuits	0.28±0.36	0.00-3.00
Corn chips, potato crisps	0.10±0.13	0.00-1.00

^a^Daily Equivalent Frequency

^b^Fruit recommendations based on 2 serves / day and vegetable recommendations based on 5 serves / day

### Physical activity

Physical activity levels are presented in Table 3. Overall, mean total PA time was 350.94 ± 281.10 min/week. Average time spent walking (251.97 ± 196.78 min/week) was greater than time spent in moderate (36.68 ± 88.58 min/week) or vigorous activity (61.74 ± 109.96 min/week). Overall, 63.2 % of women met PA recommendations defined as ≥150 minutes per week plus ≥ 5 sessions per week [50].

**Table 3:** Maternal postpartum physical activity patterns and sedentary behaviours

	n	Mean ± SD
Postpartum physical activity (min/week)		
Total	445	350.94±281.10
Walking	447	251.97±196.78
Moderate	446	36.68±88.58
Vigorous	448	61.74±109.96
Meeting weekly PA recommendations^a^ (n=446)	n	(%)
Yes	282	63.2
No	155	34.8
Women classified as sedentary	9	2.0

^a^ Recommendations based on ≥150 minutes per week plus ≥ 5 sessions per week

### Clinician advice

Table 4 shows the proportion of women who reported receiving diet and physical activity advice during pregnancy and during the postpartum period, and associations of provision of advice during the postpartum period with maternal health outcomes at 3 months postpartum. A significantly higher proportion of women reported they had received advice for healthy eating during pregnancy (87.1 %) compared with during the postpartum period (47.5 %) (p < 0.01). Likewise, a significantly higher proportion of women reported receiving physical activity advice during pregnancy (82.8 %) compared with during the postpartum period (51.9 %) (p < 0.01). There was no significant association between the mothers being provided with dietary advice and meeting fruit or vegetable intake, soft drink or non-core food intake recommendations. For physical activity, no significant association was found between provision of clinician advice to be physically active and total physical activity time, time spent walking, or whether the women met physical activity recommendations.

**Table 4:** Associations of advice received with maternal postpartum diet and physical activity

	n	(%)		n	(%)		β-coef (95%CI)	p-value	OR/RRR (95%CI)	p-value
Advice during pregnancy			Advice since delivering this baby^a^							
Eating a healthy diet?			Eating a healthy diet?							
Yes	390	87.1	Yes	213	47.5	Fruit meeting recommendations			0.91(0.58,1.43)	0.668
No	58	12.9	No	235	52.5	Veg meeting recommendations			0.69(0.31,1.54)	0.363
						Soft drink intake (glasses)				
						< one glass			1.21^b^ (0.75,1.97)	0.432
						≥ one glass			1.58^b^ (0.80,3.11)	0.192
						Non-core snack food (DEF score)	0.05(−0.03,0.13)	0.225		
Being physically active?			Being physically active?			Physical activity (min/week)				
Yes	368	82.8	Yes	231	51.9	Total time Walking	0.50 (−0.99,2.00)	0.504		
No	77	17.2	No	213	48.1	PA meeting recommendations	0.16 (−1.03,1.35)	0.791	1.08 (0.73, 1.60)	0.702

^a^Analyses controlled for maternal age, education, income, postpartum BMI and / or GWG and clustering by first time mother’s group

^b^where soft drink intake ‘none’ was used as the reference category

## Discussion

This study showed that relatively few women received advice regarding diet and physical activity in the period between childbirth and 3 months postpartum. It also showed that advice during the postpartum period was less commonly received compared with during pregnancy, and that practitioner advice was not associated with healthy postpartum diet and physical activity behaviours in this sample of first time mothers.

Importantly diet and physical activity levels in this sample were inadequate overall. Only half of the women in our study were meeting recommended intakes for fruit and less than 10 % for vegetables or combined fruit/vegetable intakes. The small number of previous studies that have assessed dietary intake in mothers, have similarly shown diet quality to be suboptimal for women during the postpartum period [14, 17]. From a public health perspective this is concerning given the well documented health benefits associated with optimal intakes with reduced chronic disease risk [8]. Therefore strategies which assist in promoting adequate fruit and vegetable intakes in mothers are vital to ensure optimizing maternal health and wellbeing in the short and long term.

On average, time spent walking was much greater than time spent in both moderate and vigorous physical activity amongst women in this study. This is not surprising, as walking has been previously found to be the most common form of physical activity for new mothers [58] and is a highly suitable form of physical activity for this population group. Walking is functional, low cost and low risk [59]. Further, the health benefits associated with walking are particularly relevant to new mothers. For example, in the U.S. an intervention study conducted by Davenport et al., [60] examined the effect of postpartum exercise intensity on chronic disease risk factors. They found that risk factors for chronic disease, including BMI, were significantly lower for women in the walking intervention groups, compared women in the control group [60]. Furthermore, there was no additional benefit seen for women in the higher intensity walking group, suggesting that even low intensity walking on most days of the week can be beneficial for the health of a new mother during the postpartum period. Elsewhere, achieving ≥30 minutes of walking per day has been associated with the prevention of weight retention at 1 year postpartum [25].

The results from our study showed that approximately two thirds of women engaged in physical activity which met recommendations defined as 150 min/week over ≥5 sessions, consistent with the 1999 national physical activity recommendations for Australian adults [61]. It should be noted, however that the more recently developed national physical activity guidelines in from 2014, differ slightly to the former recommendations. Regardless, promoting physical activity during the postpartum period is an important consideration both for greater chance of supporting reductions in PPWR, when combined with dietary strategies [31, 62], as well as assisting in the reduction of chronic disease risk.

Overall, just less than half of the sample of women in this study reported having received any dietary advice by 3 months postpartum, despite women having been shown to be particularly receptive to dietary information during the postpartum period [63]. Women have been described as being an ideal population for receiving nutrition education (149) and in the U.S., for example, the Academy of Nutrition and Dietetics and the American Society for Nutrition recently recommended that women of reproductive age receive counselling on the importance of healthy eating including during the postpartum period [12, 64]. It may be that a lack of formal guidelines in Australia addressing dietary advice for new mothers might in part explain why many women had not received such education by 3 months postpartum.

Furthermore, almost half of the women in this study reported that they had not received advice about physical activity by 3 months postpartum. This is consistent with findings from elsewhere, whereby many women have reported not received advice at all to be physically active during the postpartum [65–67]. For example, in the U.S, Ferrari et al. (2010) assessed clinician’s provision of physical activity advice to women at 3 months postpartum [65] and found that the majority of women (89.1 %) reported receiving no physical activity advice (77.4 %) [65]. This omission of advice occurred against a backdrop of Institute of Medicine recommendations that physical activity counselling be included in postpartum care for all women [68], regardless of weight status. Similarly, in the UK national guidelines recommend that women are encouraged to keep a healthy diet and be physically active during and after pregnancy [38].

Moreover, the proportion of women who reported receiving both healthy eating advice and advice to be physically active was far less during the postpartum period compared with during pregnancy. In part, this might be due to less opportunity for women to see a healthcare practitioner during the postpartum period, for their own health as a focus, compared with during pregnancy. In the months following childbirth, a shift in focus from the health of the woman to the health of the newborn is not uncommon, making the opportunity for practitioners to engage with women regarding diet and physical activity during this time to be infrequent. Whilst it may be assumed that practitioner advice may lead to women adopting healthy behaviours, interestingly, this study showed that when advice was received, it did not predict healthy diet and physical activity behaviours. Likewise, a growing number of antenatal interventions have compared information provision (typically a brochure or meeting with health professional) with additional behaviour change strategies (such as goal setting and behavioural monitoring) and found that the information provision has very little or no impact on dietary behaviours [69] or physical activity [70]. Yet, other studies have shown positive associations of provision of weight advice during pregnancy, for example, with pregnancy weight gain [71, 72].

Provision of information alone may not be sufficient to change behaviour given the many potential barriers women face to both eating healthily and being sufficiently active during and after pregnancy, including lack of time, motivation, and prioritizing their own healthy eating secondary to the demands of having a child [33]. Strategies which account for unique barriers faced by women in the postpartum period are required. Barriers such as a lack of partner support, mothers returning to work, difficulties with childcare options and strong social expectations of the role of a new mother are also common and should be taken into account when planning supportive strategies to assist new mothers in optimizing healthy behaviours [16, 73, 74].

In addition, perhaps alternative strategies to the mere provision of advice might be more effective in engaging postpartum women and promoting healthy behaviour change. For example, current evidence suggests that women should be supported to self-monitor their weight or set and review behavioural goals in an effort to successfully change behaviours such as increasing physical activity [75, 76]. Techniques identified by research elsewhere during pregnancy, which have assisted healthy weight gain have included self-monitoring of behaviour, women being provided with information related to the consequences of behaviour to the individual and providing rewards contingent on successful behaviour, as well as motivational interviewing [77]. Nonetheless, from a public health perspective, there appears to be a need to support clinicians, through alternate strategies such as lifestyle intervention programs targeting improved diet and physical activity behaviours amongst postpartum women.

A strength of this study was the assessment of frequency of advice both related to diet and physical activity. Assessing provision of advice related to both lifestyle components is important, as it has previously been shown that lifestyle changes, including both dietary intake and physical activity in combination, are more likely to be successful in minimizing PPWR for example, rather than one behavior alone [31, 62, 78, 79]. Therefore understanding predictors of these behaviours is key to addressing opportunities for behavior change. A further strength of this study was comparison of the reported advice received during and following pregnancy. This comparison allows identification of gaps in current management strategies to be made, and showed that the postpartum period can be considered a missed opportunity at present, for provision of healthy lifestyle advice, thereby supporting the need for greater provision of support in the months following childbirth.

The main limitation of this study was the inability to determine what specific advice regarding weight, diet or physical activity was provided to women. This is an important consideration, for example regarding gestational weight gain, as when healthcare practitioners do use target weights for women as part of their practice, studies have shown that women often adhere to guidelines [71, 80, 81]. Provision of non-specific advice, such as clearly defined fruit and vegetable recommendations or physical activity recommendations, may have been one reason why no associations with advice and healthy lifestyle behaviours was seen, yet this is unable to be determined. Further, this sample of women were predominately highly educated and over half of the sample of women had moderate to high household income therefore limiting the generalizability of the results. Future research would ideally consider practitioner lifestyle advice provided to low income women to enable specific delivery of postpartum support amongst different population groups following childbirth.

## Conclusion

This study showed that women reported receiving healthy eating and physical activity advice far less during the postpartum period, compared with during pregnancy. Moreover, when advice was provided to new mothers, it was not shown to be associated with healthy lifestyle behaviours. Additional investigation is warranted to identify effective forms of practitioner engagement to promote women’s diet and physical activity during the months following childbirth, to enable women to achieve optimal diet and physical activity habits for their own health benefit. Strategies which employ more intensive support, such as lifestyle interventions, tailored specifically to new mothers, as opposed to diet and physical activity advice only, is an important consideration for future research.
